# Transgenic *ZmMYB167 Miscanthus sinensis* with increased lignin to boost bioenergy generation for the bioeconomy

**DOI:** 10.1186/s13068-023-02279-2

**Published:** 2023-02-22

**Authors:** Rakesh Bhatia, Emma Timms-Taravella, Luned A. Roberts, Odin M. Moron-Garcia, Barbara Hauck, Sue Dalton, Joe A. Gallagher, Moritz Wagner, John Clifton-Brown, Maurice Bosch

**Affiliations:** 1 Institute of Biological, Environmental and Rural Sciences (IBERS), Aberystwyth University, Plas Gogerddan, Aberystwyth, SY23 3EE UK; 2 Department of Agronomy and Plant Breeding, Justus Liebig University Giessen, Heinrich-Buff-Ring 26-32, 35392 Giessen, Germany; 3 Department of Applied Ecology, Geisenheim University, Geisenheim, Germany

**Keywords:** Bio-based economy, Bioenergy, Biomass, Cell wall, Genetic engineering, Lignin, Lignocellulose, *Miscanthus sinensis*, MYB transcription factor, Transgenics

## Abstract

**Background:**

Perennial C_4_ grasses from the genus *Miscanthus* are widely regarded as leading and promising dedicated bioenergy crops due to their high biomass accumulation on marginal land with low environmental impacts and maintenance requirements over its productive life. There is an urgent socio-political and environmental need to ramp up the production of alternative, affordable and green bioenergy sources and to re-direct the net zero carbon emissions trajectory. Hence, up-scaling of *Miscanthus* cultivation as a source of biomass for renewable energy could play an important role to strategically address sustainable development goals for a growing bio-based economy. Certain *Miscanthus sinensis* genotypes are particularly interesting for their biomass productivity across a wide range of locations. As the aromatic biomass component lignin exhibits a higher energy density than cell wall polysaccharides and is generally used as an indicator for heating or calorific value, genetic engineering could be a feasible strategy to develop *M. sinensis* biomass with increased lignin content and thus improving the energetic value of the biomass.

**Results:**

For this purpose, transgenic *M. sinensis* were generated by *Agrobacterium-*mediated transformation for expression of *ZmMYB167*, a MYB transcription factor known for regulating lignin biosynthesis in C_3_ and C_4_ grasses. Four independent transgenic *ZmMYB167 Miscanthus* lines were obtained. Agronomic traits such as plant height, tillering and above-ground dry weight biomass of the transgenic plants were not different to that of wild-type control plants. Total lignin content of the transgenic plants was ~ 15–24% higher compared with control plants. However, the structural carbohydrates, glucan and xylan, were decreased by ~ 2–7% and ~ 8–10%, respectively, in the transgenic plants. Moreover, expression of *ZmMYB167* in transgenic plants did not alter lignin composition, phenolic compounds or enzymatic saccharification efficiency yields but importantly improved total energy levels in *Miscanthus* biomass, equivalent to 10% higher energy yield per hectare.

**Conclusions:**

This study highlights *ZmMYB167* as a suitable target for genetic lignin bioengineering interventions aimed at advancing and developing lignocellulosic biomass supply chains for sustainable production of renewable bioenergy.

**Supplementary Information:**

The online version contains supplementary material available at 10.1186/s13068-023-02279-2.

## Background

The status quo of attaining the net zero carbon emissions trajectory for bioenergy generation by 2030 has recently been altered from being on track to requiring more effort, as the average annual growth of bioenergy generation (8 GW) has been below the necessary level (15 GW per year) over the last five years [1]. There is growing demand and interest to use biomass as a renewable carbon resource for the bioenergy sector because it offers a transitional solution for co-firing at existing coal power stations that still have a long economic lifetime, while other technologies for dedicated biomass heating boilers and anaerobic digestion are still at the scale-up stage. The dedicated bioenergy crop *Miscanthus*, a perennial non-grain giant grass exhibiting C_4_ photosynthesis and a close relative of sugarcane (*Saccharum* spp), is a promising renewable resource that could contribute toward achieving net zero carbon emissions and ramping up the production of green energy due to its low environmental footprint, ecological benefits and high biomass yields per hectare in various climates and marginal lands [2, 3].

Currently, *Miscanthus* × *giganteus* (*M* × *g*), a sterile interspecific hybrid of a cross between *Miscanthus sacchariflorus* and *Miscanthus sinensis*, is the only commercial genotype that is widely cultivated in the UK and Europe as a valuable commodity for farmers, with a growing period of 15–20 years and harvestable yields ranging from 10 to 25 t/ha of dry matter [4, 5]. Such variable dry matter yields of *M* × *g* are related to soil effects (clayey, sandy and loamy), along with its sensitivity to low temperatures, heavy frost and lack of water availability during droughts. Considering these physiological disadvantages and some of the current commercial expansion limitations associated with *M* × *g* such as low cloning rates and high biomass production costs, *M. sinensis* represents an alternative and one of the most favourable *Miscanthus* species because it shows high genetic diversity ideal for breeding new *Miscanthus* varieties and more water and cold tolerance, thereby producing annually more stable and high lignocellulosic biomass yields [6].

The gross make-up of *Miscanthus* lignocellulosic biomass comprises three major structural polymers cellulose, hemicellulose and lignin and there is extensive variation in biomass composition between different *Miscanthus* species and genotypes, harvest periods (autumn and winter) and tissue type (stem and leaf), with cellulose ranging from ~ 20 to 52%, hemicellulose from ~ 23 to 36% and lignin from ~ 9 to 31% of dry matter [7–9]. The linear cellulose and branched hemicelluloses (such as arabinoxylan and mixed-linkage glucan) are the main polysaccharides that form the structural framework of plant lignocellulosic biomass. Lignin, a complex polyphenolic polymer and an integral part of lignocellulose, is composed of three canonical monolignol subunits, syringyl (S), guaiacyl (G) and *p*-hydroxyphenyls (H), which vary substantially depending on the biomass feedstock [10]. Apart from the three major lignin monomers, *Miscanthus* incorporates hydroxycinnamates such as ferulic acid (FA) and *p*-coumaric acid (*p*-CA) that are involved in cross-linking the polysaccharides lignin complex, with FA usually attached to arabinoxylans while *p*-CA is predominantly bound to lignin with minor amounts also attached to arabinoxylans [11].

The relative abundances, cross-linkages, interactions and arrangements of these lignocellulosic components collectively form the basis of biomass recalcitrance and play a crucial role in the processing efficiency for the production of renewable and sustainable biofuels and commodity bio-based products [12, 13]. For instance, considering the industrial process requirements to produce bioenergy for end-use, *Miscanthus* biomass with high lignin and low ash content are preferred traits for thermochemical processes (combustion, pyrolysis or gasification), whereas high cellulose and low lignin content are appealing biomass qualities for biochemical processes (enzymatic hydrolysis and fermentation to liquid biofuels) [14, 15].

One of the ways to inherently improve the biomass of C_4_ grasses for bioconversion processes involves genetically modifying the expression levels of proteins that function as transcription factors (TF) [16, 17]. The MYB (MYELOBLASTOSIS) family of proteins, one of the largest plant-specific TF families, can bind to the regulatory elements (enhancers and silencers) of target genes and thus play a key role in regulating (activate or repress) the transcription of specific target genes related to the biosynthesis of lignocellulose, thereby affecting biomass composition and accumulation. For instance, in transgenic sorghum plants, over-expression of the transcriptional regulator *SbMYB60* was associated with increased lignin biosynthesis and higher energy content of the biomass [18]. Conversely, over-expression of *PvMYB4* in switchgrass reduced lignin content and improved cellulosic ethanol yield [19, 20]. However, these studies showed transgenic plants with altered growth phenotypes, a common feature of transgenic approaches aimed at adapting and designing biomass for various conversion processes. In contrast, no negative phenotypic drawbacks were observed for engineered maize plants with increased lignin amounts through over-expression of *ZmMYB167* [21], previously identified as a promising MYB candidate for the transcriptional regulation of secondary cell wall biosynthesis in grasses [22]. Even though an *Agrobacterium*-based transformation strategy for *M. sinensis* was recently described with the potential to improve biochemical processes and bioethanol production [23], the C_4_ grass *Miscanthus* has been less explored for genetic improvements of the biomass for thermochemical processes.

The goal of this present study was to redesign the biomass composition of *M. sinensis* for improved heating or energy value using the TF-based genetic engineering strategy. For this, transgenic lignin-rich *M. sinensis* biomass was bioengineered via the expression of the TF gene *ZmMYB167*, known as a potential transcriptional regulator of lignin biosynthesis, based on initial work in the two genetic grass model systems *Brachypodium distachyon* and *Zea mays* [21]. Results show that the biomass from these high-lignin *ZmMYB167 M. sinensis* plants exhibited an increased energy content, without an enzymatic saccharification efficiency or above-ground biomass yield penalty.

## Results and discussion

### Expression of ZmMYB167 in *Miscanthus*

**Fig. 1:**
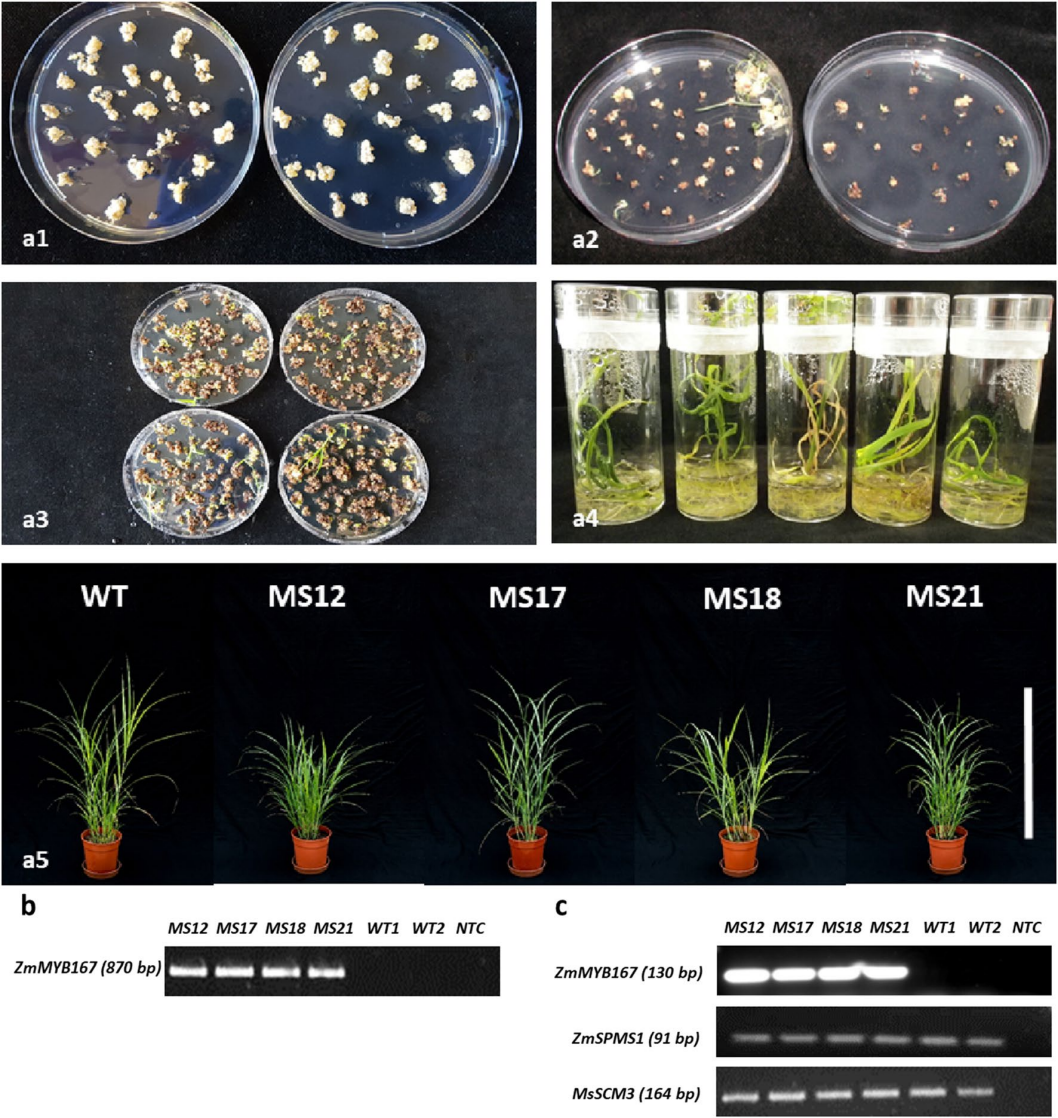
Expression of *ZmMYB167* in transgenic *Miscanthus sinensis*. **a** Representative images of the *Miscanthus* transformation procedure. a1, embryogenic callus used for transformation; a2, transformed callus on selection medium; a3, regenerated plantlets on regeneration medium; a4, regenerated plantlets on rooting medium; a5, regenerated *ZmMYB167* plants from four independent *ZmMYB167* transgenic *Miscanthus* at the leaf and stem development stage, scale bar = 1 m. **b** genomic DNA PCR analysis of *ZmMYB167* transgenic and wild-type control plants using primers covering the *ZmUbi1* promoter region and the *ZmMYB167* transgene-specific region. **c** RT-PCR analysis of *ZmMYB167 Miscanthus* using *ZmMYB167*, *ZmSPMS1* and *MsSCM3* gene-specific primers. *MS* transgenic line; *WT* wild-type; *NTC* no template control

**Fig. 2:**
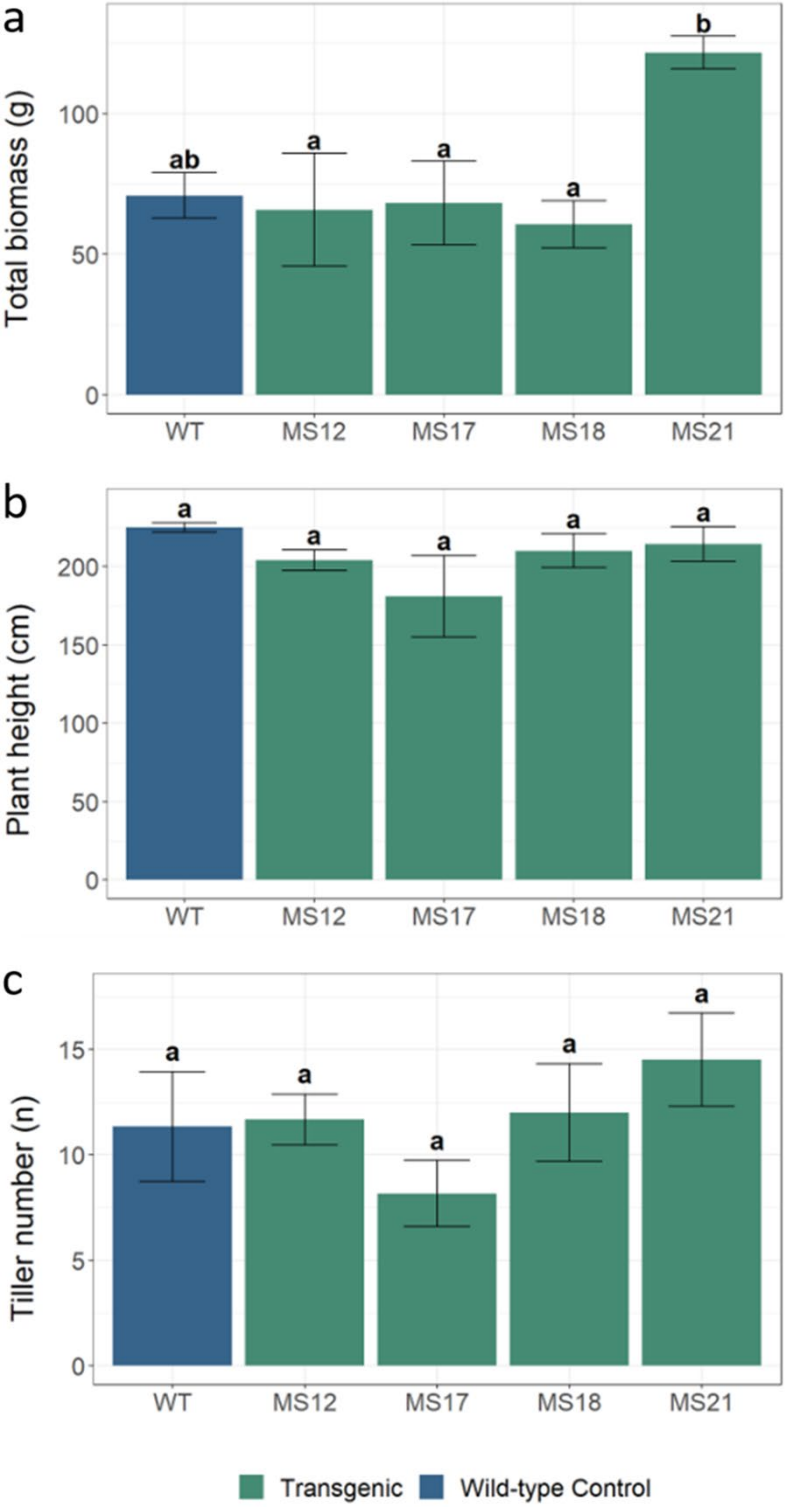
Phenotypic traits of the wild-type control and *ZmMYB167 Miscanthus* plants. Agronomic characteristics at the senescence stage. **a** Biomass accumulation is expressed as total dry above-ground biomass. **b** Plant height measured from base to tip of the inflorescence of the tallest tiller of fully senesced plants. **c** Tiller count is recorded as the number of stems per plant. Data are means ± standard error of at least five transgenic plants (*n* ≥ 5). For wild-type controls, *n* = 3. Different letters within each plot indicate significant differences (*P* ≤ 0.05) following a one-way ANOVA with a post hoc Tukey test

To substantiate *ZmMYB167* association with lignin biosynthesis and as a target for biotechnology to improve bioenergy grass feedstock, four independent transgenic *M. sinensis* lines (named MS12, MS17, MS18 and MS21) harbouring *ZmMYB167* under the control of *ZmUbi1* and exhibiting heterologous expression of the *ZmMYB167* transcript (Fig. 1) were produced and evaluated for plant fitness and biomass characteristics. The expression level of *M. sinensis MsSCM3* [24], a putative orthologue of ZmMYB167, was not affected by the heterologous expression of *ZmMYB167* (Fig. 1), suggesting that cross-regulation did not occur *in planta*. The *ZmMYB167 Miscanthus* expression lines showed no plant growth defects at the senescence stage (Fig. 2). Although there was variation in biomass accumulation (~ 60–120 g), plant height (~ 170–210 cm), and tillering (~ 8–13) for the transgenic *Miscanthus* plants, these phenotypic traits were not statistically different from their wild-type controls, which can have implications for the development of transgenic *Miscanthus* as an alternative, renewable and sustainable lignocellulosic biomass resource. However, the fitness and biomass traits of the lignin-modified transgenic *ZmMYB167 Miscanthus* plants require careful evaluations in real-world field conditions over several growing seasons, as follow-up field trials with transgenic *PvMYB4* switchgrass plants showed that only 3/8 of transgenic lines planted out survived the first field winter and that only 1/8 of transgenic line exhibited increased biofuel production traits over two growing seasons [25].

### Compositional analysis of transgenic *ZmMYB167 Miscanthus*

To evaluate the impact of *ZmMYB167* expression on biomass characteristics, a series of chemical and structural analyses were performed on transgenic *ZmMYB167 Miscanthus* and wild-type plants. All four *ZmMYB167* transgenic *Miscanthus* lines exhibited increased levels of Klason lignin content by ~ 15–24% compared with wild-type controls (Table 1). Such increases in lignin content paralleled those of other MYB over-expression studies targeting the transcriptional activation of lignin biosynthesis [26, 27]. The overall abundance of the two main structural cell wall polysaccharides, glucan and xylan, were concomitantly decreased by ~ 2–7% and ~ 8–10%, respectively (Table 1). Similar compositional results were observed for sorghum *SbMYB60* over-expression events [18]. The increase in lignin and decrease in structural carbohydrates is probably a consequence of redirecting the cell wall biosynthesis carbon sink and flux away from polysaccharides and to the monolignol biosynthesis pathway, providing further evidence of *ZmMYB167* role in potentially regulating and inducing lignin biosynthesis. It is also possible that the expression of *ZmMYB167* could have stimulated the activation and/or repression of other MYBs or major plant TF families (AP2/ERF or NAC) within the cell wall biosynthesis TF network [28, 29], thereby co-regulating cellulose and hemicellulose biosynthesis and reducing structural carbohydrates (glucan and xylan) amounts in the transgenic *Miscanthus* lines Since *ZmMYB167* expression was shown to dramatically increase the amount of Klason lignin in *Miscanthus*, lignin composition and the relative abundances of the two main lignin biosynthesis pathway intermediates (*p*-CA and FA) which form cross‐linkages between polysaccharides and lignin were also measured (Table 2). Interestingly, the relative percentage of S and G lignin monomers, and hence the S/G ratio, were generally not statistically different between the *Miscanthus ZmMYB167* expression lines and controls (except for MS 18) (Table 2). In addition, *p*-CA and FA levels (varying between ~ 3 and 12 mg/g dry weight cell wall residue (CWR)) were similar to previously reported data [11, 30]. The amount of *p*-CA was only significantly elevated in biomass collected from MS17 and MS21, while FA levels were only elevated in biomass collected from MS18 (Table 2). These results collectively suggest that *ZmMYB167* expression appears to have primarily driven the flux towards biosynthesis of monolignols and incorporation of lignin into *Miscanthus* biomass.

**Table 1:** Structural carbohydrates and lignin in transgenic *ZmMYB167 Miscanthus* plants on an extractive-free basis

*Extractives free (w/w % of DM solids)*						
*Miscanthus*	Glucan	Xylan	Arabinan	Galactan	Acetyl	Klason lignin
WT	40.27 ± 0.77^b^	28.61 ± 0.46^b^	2.75 ± 0.03^ab^	0.51 ± 0.01^a^	5.93 ± 0.29^a^	21.94 ± 0.96^b^
MS12	39.34 ± 0.31^ab^	26.12 ± 0.29^a^	2.68 ± 0.06^a^	0.54 ± 0.02^a^	6.03 ± 0.13^a^	25.29 ± 0.32^a^
MS17	37.59 ± 0.48^a^	25.72 ± 0.24^a^	2.60 ± 0.08^a^	0.52 ± 0.02^a^	6.29 ± 0.12^a^	27.28 ± 0.30^a^
MS18	37.98 ± 0.41^a^	26.21 ± 0.33^a^	2.95 ± 0.05^b^	0.63 ± 0.03^b^	6.16 ± 0.08^a^	26.06 ± 0.63^a^
MS21	38.43 ± 0.36^ab^	25.93 ± 0.48^a^	2.65 ± 0.07^a^	0.55 ± 0.02^a^	5.69 ± 0.22^a^	26.76 ± 0.36^a^

Data are means ± standard error of at least two experimental replicates from two plants per event. Klason lignin is total lignin (acid-soluble and acid-insoluble lignin). Extractives content was not determined. Data were normalised to a summative mass closure of 100%. Different letters indicate significant differences (*p* ≤ 0.05) following a one-way ANOVA with a post hoc Tukey test

**Table 2:** Analysis of lignin composition and phenolic acid content in stems of transgenic *ZmMYB167 Miscanthus* plants

*Miscanthus*	Lignin-derived thioacidolysis monomers				Ester-linked phenolic acids (mg/g CWR)	
	H (%)	G (%)	S (%)	S/G ratio	*p*-CA	FA
WT	5.52 ± 0.33^a^	59.66 ± 0.49^a^	34.82 ± 0.53^b^	0.58 ± 0.01^b^	9.59 ± 0.25^c^	3.02 ± 0.06^b^
MS12	4.82 ± 0.42^a^	56.67 ± 0.90^a^	38.51 ± 1.27^ab^	0.68 ± 0.03^ab^	9.86 ± 0.29^c^	3.02 ± 0.12^b^
MS17	5.43 ± 0.72^a^	59.97 ± 1.10^a^	34.60 ± 1.51^ab^	0.58 ± 0.04^ab^	11.08 ± 0.37^ab^	3.19 ± 0.05^ab^
MS18	4.69 ± 0.43^a^	55.49 ± 0.88^a^	40.33 ± 1.30^a^	0.73 ± 0.04^a^	10.60 ± 0.23^ac^	3.49 ± 0.08^a^
MS21	4.98 ± 0.43^a^	56.69 ± 1.55^a^	38.33 ± 1.61^ab^	0.69 ± 0.05^ab^	11.84 ± 0.19^b^	2.97 ± 0.05^b^

Data are means ± standard error of three technical replicates from two individual plants per event. Different letters indicate significant differences (*p* ≤ 0.05) following a one-way ANOVA with a post hoc Tukey test

*H p*-hydroxyphenyl; *G* guaiacyl; *S* syringyl. *WT* wild-type. *CWR* cell wall residue

**Fig. 3:**
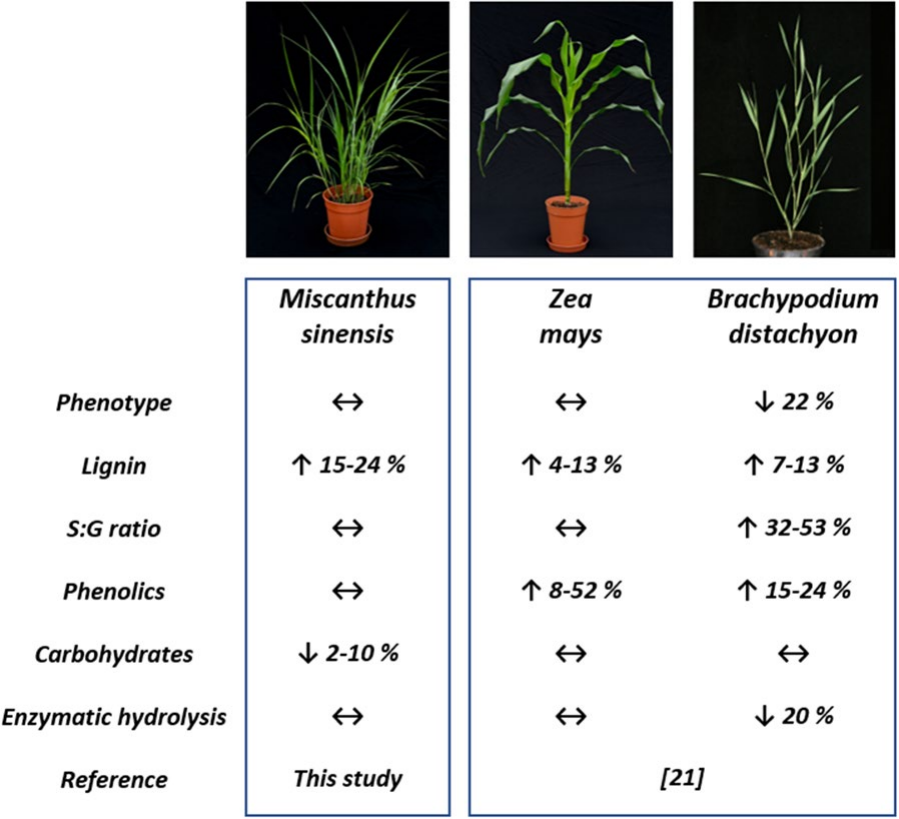
Agronomic and lignocellulosic characteristics for *ZmMYB167* expression in *Miscanthus sinensis*, *Zea mays* and *Brachypodium distachyon. ↑ increase, ↓ decrease, ↔ no change*

A previous study showed that increasing the *ZmMYB167* expression levels in *Zea mays* and its heterologous expression in *Brachypodium* likewise resulted in increased lignin content by ~ 4–13% as well as higher abundances of *p*-CA and FA levels by ~ 8–52%, and in the case of *Brachypodium*, also resulted in increases in S/G ratio by 32–53%, while no significant changes in polysaccharides content were detected for both the grass model species [21]. Furthermore, the growth and development of transgenic *ZmMYB167 Brachypodium* plants were shown to be impaired and similar elevations in lignin content in transgenic *SbMYB60* sorghum were previously shown to have reduced plant fitness likely due to lignin deposition impeding cell wall expansion during growth [18]. A summary of the agronomic and lignocellulosic biomass characteristics related to the expression of *ZmMYB167* in *Miscanthus sinensis*, *Zea mays* and *Brachypodium distachyon* is highlighted in Fig. 3. Despite the differences in biomass composition and phenotypes, similarities and consistency in the elevations in lignin content for the *M. sinensis*, *Zea mays* and *B. distachyon ZmMYB167* over-expression lines may substantiate the ZmMYB167 TF as a monolignol biosynthesis regulator in grasses. Taken together, these results highlight the opportunities of using TFs such as ZmMYB167 as a genetic engineering tool to alter biomass composition but also emphasises the difficulties in predicting outcomes of such transgenic approaches in grasses, and the need to better understand gene targets and protein–protein interactions of TFs regulating cell wall biosynthesis as well as the metabolic fluxes through the cell wall biosynthesis pathways in the C_3_ and C_4_ grasses [17].

### FTIR analysis and enzymatic hydrolysis of transgenic ZmMYB167 *Miscanthus*

**Fig. 4:**
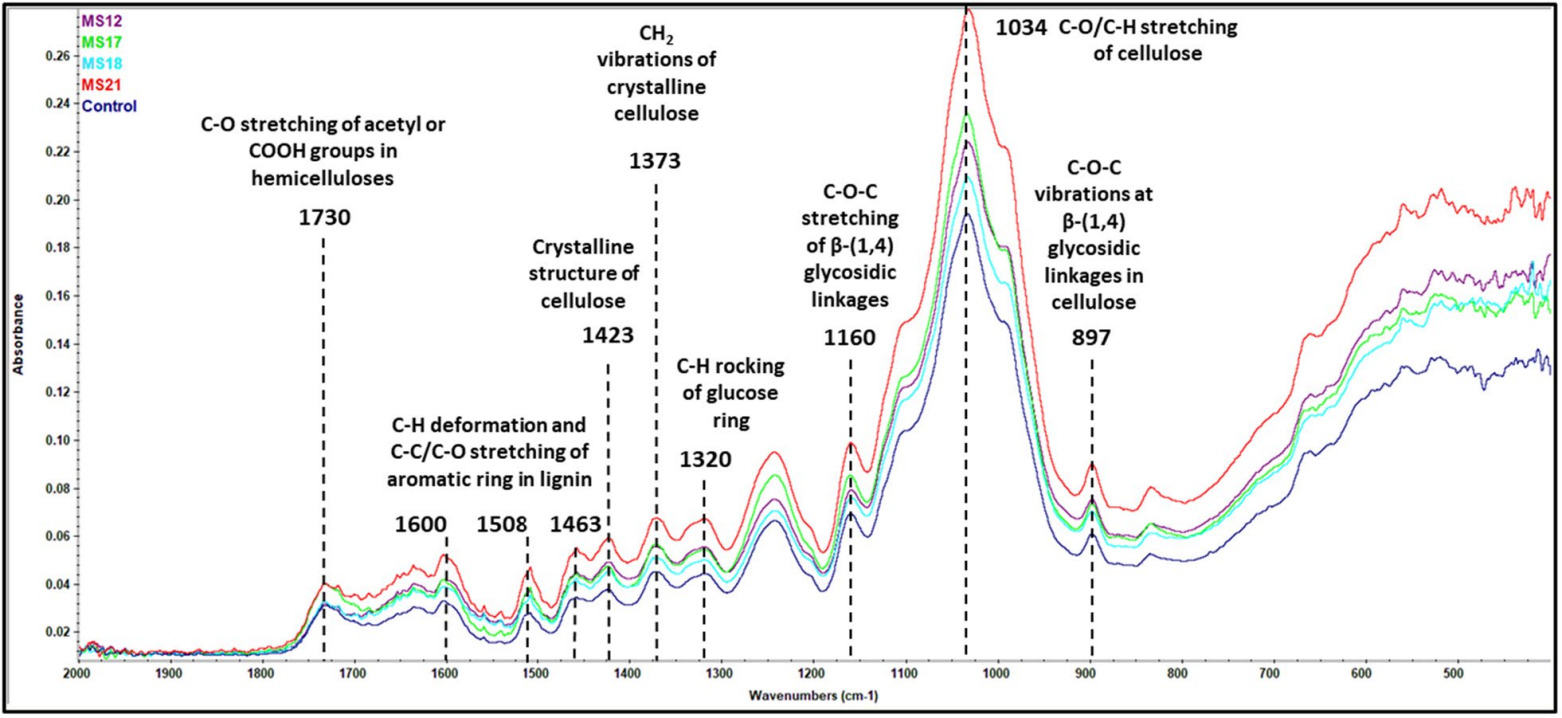
FTIR spectra of the wild-type control and *ZmMYB167* transgenic *Miscanthus* plants

To further evaluate the impact of *ZmMYB167* expression on structural biomass features, transgenic *Miscanthus* lines were analysed by Fourier-transform infrared (FTIR) spectroscopy which is a rapid, non-invasive and high-throughput tool to study arrangements of biomass components as well as cross-linkages [31]. Figure 4 shows the FTIR spectra of the wild-type control and *ZmMYB167* transgenic *Miscanthus* plants with band assignments according to the literature [32–34]. Peaks in the 890 cm^−1^–1430 cm^−1^ region are associated mainly with the presence of the structural carbohydrate glucan. Several peak ratios such as that for 1423 and 897 cm^−1^ related to the cellulose crystallinity or lateral order index (LOI) were significantly higher in all transgenic plants compared with the control (Additional file 1: Table S1). This FTIR result suggests that the glucan structure in *ZmMYB167 Miscanthus* comprises a more highly ordered form compared to the wild-type plants [32], and is likely reflective of the ~ 2–7% glucan reduction in the transgenic lines (Table 1). Moreover, the hydrogen bond intensity (HBI) from the peak ratio at 3400 and 1320 cm^−1^, closely related to the degree of hydrogen bonding between the glucan crystalline system, was generally not significantly altered in the transgenics (except for MS12) compared with control plants (Additional file 1:Table S1). This could be attributed to the fact that expression of *ZmMYB167* and the increase in lignin content may have also led to a reorganisation of the glucan assembly without affecting the HBI between neighbouring glucan chains possibly due to a less packed glucan structure.

The lignin region (1460–1600 cm^−1^) intensified in the FTIR spectra (Fig. 4) for the four transgenic *ZmMYB167* plants and these modifications may correspond to extensive lignification of the biomass [35]. However, the cross-linked lignin (CLL) ratio (1600 and 1508 cm^-1^) that evaluates the proportion of lignin with condensed and cross-linked structures [32], was significantly increased only for the MS12 and MS17 transgenic lines (Additional file 1: Table S1), indicative of enrichment in lignin with cross-linked FA or* p*-CA. Then again, the 1730 cm^−1^ peak intensity in Fig. 4 attributed to the degree of xylan acetylation or esterification of the carboxylic group of the FA and* p*-CA with lignin and/or xylan [36, 37] is suggestive of higher esterification for the transgenic lines MS17 and MS21 and appears consistent with the higher *p*-CA contents (Table 2). In summary, the FTIR results highlighted in Fig. 4 support and concur with the chemical analyses and suggests that expression of *ZmMYB167* leads to compositional and structural modifications of the biomass in the transgenic *Miscanthus* lines.

**Fig. 5:**
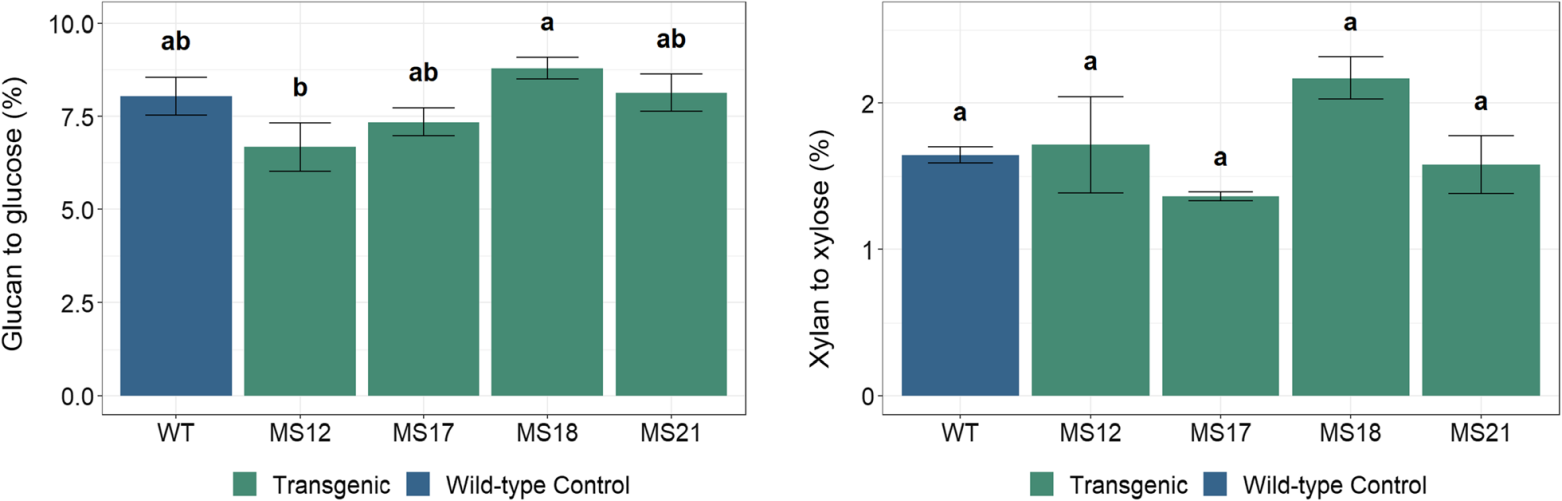
Glucose and xylose release from enzymatic saccharification of extractive-free *Miscanthus* biomass. Glucan and xylan conversion (%) to glucose and xylose, respectively, after 72 h of enzymatic hydrolysis of untreated cell wall residues obtained from transgenic *ZmMYB167 Miscanthus* relative to wild-type (WT) plants. Data are means ± standard error of three experimental replicates. Different letters indicate significant differences (*p* ≤ 0.05) following a one-way ANOVA with a post hoc Tukey test

To determine the effects of *ZmMYB167* expression on the recalcitrance of biomass and the release of glucose and xylose, the enzymatic saccharification efficiency of the CWR after 72 h using the commercially available enzyme cocktail Cellic® CTec2 was evaluated without a pretreatment step. No significant changes in enzymatically released glucose (~ 7–9%) or xylose (~ 1–2%) yields were observed between the *ZmMYB167 Miscanthus* lines and the wild-type controls (Fig. 5), a result which was comparable with the over-expression of *ZmMYB167* in the C_4_ grass *Zea mays* (Fig. 3). Lignin and the hydroxycinnamic acids FA and *p*-CA implicated in cell wall cross-linking sterically impede the accessibility of structural polysaccharides and cause cellulases to adsorb irreversibly thereby reducing the enzymatic activity during hydrolysis, negative factors that are a major concern in the pulp and paper and the bioethanol manufacturing industry [38, 39]. However, despite the changes in lignin content (Table 1) and physico-chemical properties (Fig. 3) of the transgenic *Miscanthus* lines, these did not affect the biomass recalcitrance for enzymatic saccharification efficiency (Fig. 5). There are still many unknowns concerning the relationships between the composition, structural organisation and properties of lignocellulose for the production of biomass-derived bioethanol [40]. Nonetheless, the *ZmMYB167 Miscanthus* biomass could also be a viable resource for both the valorisation of polysaccharides into fermentable sugars in the production of bioethanol and lignin into industrially relevant biofuels, materials or high value-added chemicals.

### Energy content and environmental impact analysis of transgenic ZmMYB167 *Miscanthus*

**Fig. 6:**
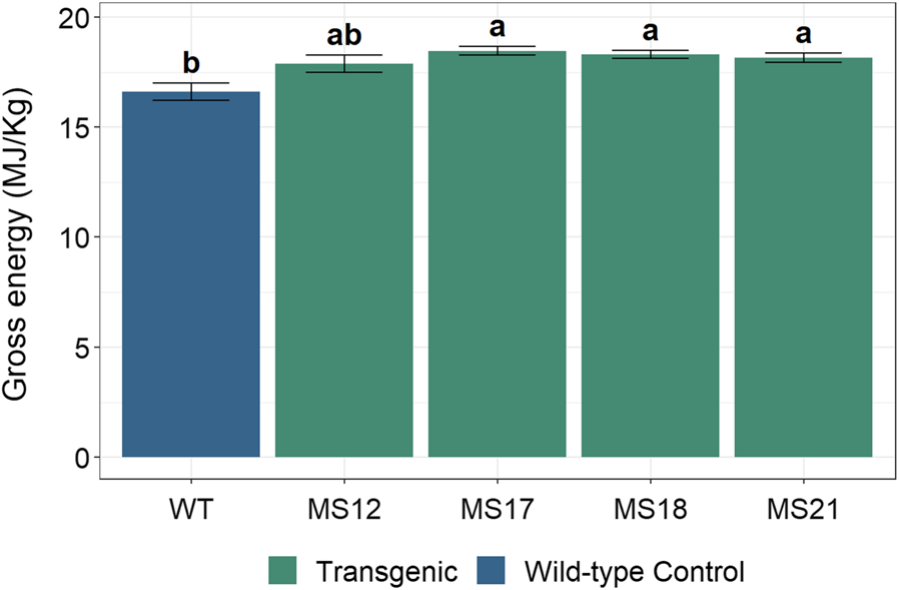
Total energy content of transgenic *ZmMYB167* and wild-type *Miscanthus* biomass. Data are means ± standard error (*n* ≥ 2). Different letters indicate significant differences (*p* ≤ 0.05) following a one-way ANOVA with a post hoc Tukey test

The combustion of *Miscanthus* biomass for heat generation can lead to considerable greenhouse gas (GHG) and energy savings compared to fossil alternatives [41]. Besides the inputs applied, the biomass yield is a crucial parameter in order to further improve the environmental performance of *Miscanthus* cultivation and utilisation [42]. Several studies have demonstrated that the use of genetically modified crops can reduce the impacts on the environment significantly through increased yields or reduced inputs [43–45]. The current study shows that the heating value of the biomass from transgenic *Miscanthus* plants increased on average by 10% (mass-based) exceeding 18 MJ/kg compared with biomass harvested from wild-type plants (Fig. 6), while at the same time maintaining biomass yields (Fig. 2). This is equivalent to a 10% higher energy yield per hectare. Assuming that the inputs needed for the cultivation process (e.g., pesticides, fertilizer, etc.) are not changing, this leads to a decrease in the impacts on the environment of the utilisation of the *Miscanthus* biomass for combustion by over 9% per kWh heat produced, thereby holistically improving the environmental performance of this utilisation pathway. In the case of bioethanol production a low- lignin content is required [46]. However, Meyer et al. [42] showed that even for this utilisation pathway the increase in lignin offers the chance to substantially reduce the global warming potential as the fermentation residues are burnt to supply process heat and electricity. A higher lignin content significantly increases the energy produced using the residues, thereby minimising the demand for energy produced with fossil fuels.

The current study shows that the use of engineered lignin-rich *Miscanthus* biomass offers the chance to improve the environmental performance of the utilisation of *Miscanthus* biomass considerably. It has to be emphasised though that potential risks associated with the environmental release of genetically modified crops are not included in the current study [47]. Furthermore, the impact of high-lignin content *Miscanthus* plants on harvest machinery at intensive levels of use and the effect on GHG emissions, energy balances and costs when harvesting this biomass residue for bioenergy production was not considered and merits additional research.

## Conclusions

There is considerable interest in the mass‐scale deployment of the perennial grass *Miscanthus* as a dedicated biomass crop and resource of sustainable and renewable bioenergy and bio-based products for the bioeconomy. However, the development and genetic improvement of *Miscanthus* cultivars to secure a sustainable biomass supply for a climate-neutral economy are hampered mostly by the long necessary plant breeding cycles and efforts. To address these challenges, plant genetic engineering represents a prominent tool that could accelerate improvements for the bioenergy crop *Miscanthus*. Strategies for generating transgenic *Miscanthus* plants are relatively new and scant compared to other routinely used crops such as maize and rice. The objective of this study was to use a biotechnological approach to produce transgenic *Miscanthus* plants that confer improved biomass traits for bioenergy conversion processes. Transgenic *M. sinensis* with higher lignin contents (~ 15–24%) were successfully bioengineered through expression of the transgene *ZmMYB167*, a known candidate for transcriptional activation of lignin biosynthesis. Results reported also show that the biomass of transgenic *ZmMYB167 Miscanthus* plants has compositional and structural alterations without an enzymatic saccharification efficiency or plant growth and development penalty. More importantly, expression of the *ZmMYB167* transgene in *Miscanthus* led to an ~ 10% increase in energy content and heating value of the biomass, thereby providing a novel route towards improving the environmental performance of *Miscanthus* cultivation and its biomass utilisation. The study draws attention to *ZmMYB167* as a target for plant biotechnology to potentially improve the next generation of the dedicated bioenergy crop *Miscanthus* and fulfil future renewable biomass supply and bioenergy needs.

## Materials and methods

### Miscanthus transformation with ZmMYB167

For the heterologous expression of *ZmMYB167* (GRMZM2G037650) in *Miscanthus sinensis*, the expression construct previously used for the expression of *ZmMYB167* in *Brachypodium distachyon* was used [21]. Briefly, *ZmMYB167* was cloned in the pIPKb002 over-expression vector [48], containing the hygromycin selection marker. The expression of *ZmMYB167* was under the control of the *ZmUBI1* promoter (Additional file 1: Fig. S1). Embryogenic callus cultures of *Miscanthus sinensis* genotype Suegen14 [49] were established from in vitro shoot tips and sub-cultured every five weeks or less for 21 weeks on WPBS-based callus induction medium as described in detail previously [50]. *Agrobacterium* strain AGL1 carrying the *ZmUBI1::ZmMYB167* expression construct was grown overnight at 25 °C on solid YEP medium containing 100 mg/L kanamycin and 25 mg/L rifampicin. A sample was added to liquid WPBS sucrose and glucose-based infection medium containing 300 μM acetosyringone and 0.02% Pluronic F68 and shaken for two hours. Finally, 2-week-old calli were transferred to the same WPBS infection medium and heat shocked in a water bath for three min at 43 °C. The *Agrobacterium* solution was added to the calli and gently mixed. The calli and *Agrobacterium* were co-cultured for 30 min before the *Agrobacterium* solution was poured off and the calli were tipped onto three filter papers in a 90 mm petri dish to dry. The calli were left to dry in the open for ~ 30 min before transfer to a fresh petri dish with three filter papers wetted with 3 mL WPBS maltose-based co-cultivation medium. The calli were left in the dark at 25 °C for three days before transfer to a WPBS sucrose-based selection medium containing 50 mg/L meropenem, 100 mg/L timentin and 50 mg/L hygromycin. After five weeks of calli culture at 25 °C in the dark, the calli from callus selection media were transferred to a WPBS regeneration medium containing the same antibiotics and grown in the light (~ 100 µE m^ − 2^ s^ − 1^) at 25 °C. Some shoots regenerated after 2 weeks and were transferred to selective WPBS liquid propagation medium with antibiotics and grown under the same conditions. Rooted plantlets were then transferred to soil and grown in a containment glasshouse under standard conditions. Natural daylight was supplemented with 16 h of artificial light (high-pressure sodium son-T plus lights). Plants were grown individually in 10-L pots, each containing John Innes No3 compost. *Miscanthus* plants of four independent transformation events designated MS12, MS17, MS18 and MS21 (T_0_ generation) were selected and used for all the analyses. *Miscanthus sinensis* genotype Suegen14 plants, grown under the same conditions, were used as wild-type controls.

### RNA isolation and PCR analysis

Developing *Miscanthus* leaves were cut at their node, dissected, and immediately frozen in liquid nitrogen. Total RNA was isolated from ground leaf samples using a combination of Trizol Reagent (Invitrogen) and the Qiagen RNAeasy Plant Mini Kit. RNA quantity was assessed using an Epoch Microplate Spectrophotometer (BioTEK). The quality and integrity of total RNA were evaluated on an agarose gel. cDNA was prepared using the SuperScript III First-Strand Synthesis SuperMix (Invitrogen) with oligo(dT) primers. PCR was performed using the Green GoTaq Reaction Buffer and DNA polymerase (Promega) with the following thermal cycling steps: 95 °C for 2 min, 35 cycles of 30 s at 95 °C, 1 min at 60 °C, and 30 s at 72 °C, final extension 72 °C for 5 min. RT-PCR products were analysed on agarose gels with *Zea mays spermine synthase1 (ZmSPMS1)* as a reference gene. The primers used for PCR amplification of genomic and complementary DNA from transgenic and wild-type control plants are listed in Additional file 1: Table S2.

### Preparation of *Miscanthus* material

Total above-ground stem biomass of fully senesced *Miscanthus* plants was prepared according to the technical reports NREL/TP-510–42620 and NREL/TP-510–42621 [51, 52]. The hammer-milled and sieved (− 20/ + 80 mesh) biomass material was then fractionated to a de-starched and extractive-free alcohol insoluble residue (AIR) according to [53, 54], dried at 45 °C per technical report NREL/TP-510–42620 [51] and stored in air-tight sealable bags until further use.

### Structural carbohydrates and Klason lignin

Compositional analysis (carbohydrates, acetyl and Klason lignin content) of *Miscanthus* AIR biomass was determined per technical report NREL/TP-510–42618 [55]. Structural carbohydrates from the concentration of the corresponding monomeric sugars were determined by High-Performance Anion Exchange Chromatography (ThermoFisher ICS-5000) coupled with Pulse Amperometric Detector (HPAEC-PAD), using the Dionex CarboPac SA10 guard (4 × 50 mm) and analytical (4 × 250 mm) columns at 45 °C, and 1 mM KOH as eluent, with an eluent flow rate of 1.5 mL/min and 25 μL injection volume. Glucose, xylose, arabinose, galactose, mannose, fructose, sucrose, cellobiose and fucose were run as calibration standards using serial dilution concentration ranges of 20 μg/mL, 10 μg/mL, 5 μg/mL, 2.5 μg/mL and 1.25 μg/mL. Monosaccharide chromatograms were analysed and processed using the Chromeleon™ 7.2 Chromatography Data System (CDS) software. The percentage of each sugar, acetyl and lignin (acid-insoluble and acid-soluble lignin) content was calculated on an oven-dry weight and extractives-free basis. Data are reported as means ± standard error (*n* ≥ 3).

### Determination of cell wall hydroxycinnamoyl esters and lignin-derived monomers

The amount of the hydroxycinnamic acid (HCA) derivatives *p*-coumaric acid (*p*-CA) and ferulic acid (FA) in *Miscanthus* AIR biomass were determined by using an alkaline saponification method as described by [56] with some modifications as described by [57]. The percentage of HCA are reported on an oven-dry weight basis. For the determination of the lignin monomer composition, *Miscanthus* AIR biomass was subjected to thioacidolysis and quantified by gas chromatography–mass spectrometry (GC–MS) analysis as described previously [21]. Data are reported as means ± standard error (*n* ≥ 3).

### Fourier-transform infrared spectroscopy (FTIR)

FTIR analysis of *Miscanthus* AIR biomass was performed using a Thermo Nicolet iS50 FTIR spectrometer [58]. Using 2 mg of milled sample (< 80 µm), FTIR spectra were recorded in duplicates and the range between 400–4000 cm^ − 1^ with a resolution of 4 cm^ − 1^ and eight scans per sample. Initial background spectra were captured and subtracted from the sample measurements before data analysis.

### Enzymatic hydrolysis of *Miscanthus*

Low solids (1% w/v) enzymatic hydrolysis of *Miscanthus* biomass was based on the technical report NREL/TP-5100–63351 [59] using Cellic® CTec2 (Novozymes A/S, Denmark) at a high enzyme dosage level of 30% w/w (g enzyme/g glucan) as outlined per the Novozymes Cellic® CTec2 application sheet for hydrolysis of lignocellulosic materials. Samples were loaded in a shaker set at 50 °C (200 rpm) and withdrawn after 72 h. The enzymatic hydrolysis was stopped by boiling samples at 100 °C for 10 min. After centrifugation (10 min, 10,000 × g), the supernatants were analysed for glucose and xylose yields by HPAEC-PAD.

### Total energy content of *Miscanthus* biomass

Energy content measurements of *Miscanthus* biomass samples were performed in a standard bomb calorimeter (Gallenkamp Autobomb, Sanyo Gallenkamp, Loughborough, UK). All samples were prepared, hammer milled and sieved (− 20/ + 80 mesh) per technical report NREL/TP-510–42620 [51], and then compressed into ~ 1 g of sample pellets using a hydraulic pelletizer. Heat content was determined by burning pellets with excess oxygen at a pressure between 25 and 30 bar in a sealed steel bomb, which is regarded as a near-adiabatic system. The bomb calorimeter was calibrated by combustion of certified benzoic acid.

### Statistical analysis

Statistical analysis was performed using the ggplots package of the R statistics software. One-way analysis of variance (ANOVA), followed by a post hoc Tukey test at *P* < 0.05, was used to compare the data between wild-type control and independent transgenic *ZmMYB167 Miscanthus* plants.

## Supplementary Information

**Additional file 1: Figure S1.** Schematic representation of the genes between left border (LB) and right border (RB) of the pIPKb002 based binary vector construct used for Agrobacterium-mediated transformation of mediated transformation of Miscanthus sinensis. **Table S1.** Cellulose and lignin-related properties in the wild-type and ZmMYB167 transgenic Miscanthus plants. **Table S2.** Sequences of primers used for detection genomic DNA and reverse transcription PCR analysis.

## Data Availability

The datasets generated and/or analysed during the current study are available from the corresponding authors upon reasonable request.
