# Supplementary material for: Transgenic ZmMYB167 Miscanthus sinensis with increased lignin to boost bioenergy generation for the bioeconomy
Source: Biotechnol Biofuels Bioprod. 2023 Feb 22;16:29. doi: 10.1186/s13068-023-02279-2 (PMC9945411; doi:10.1186/s13068-023-02279-2)
Supplement: Supplementary file 1 — Additional file 1: Figure S1. Schematic representation of the genes between left border (LB) and right border (RB) of the pIPKb002 based binary vector construct used for Agrobacterium-mediated transformation of mediated transformation of Miscanthus sinensis. Table S1. Cellulose and lignin-related properties in the wild-type and ZmMYB167 transgenic Miscanthus plants. Table S2. Sequences of primers used for detection genomic DNA and reverse transcription PCR analysis. [file 13068_2023_2279_MOESM1_ESM.pdf]

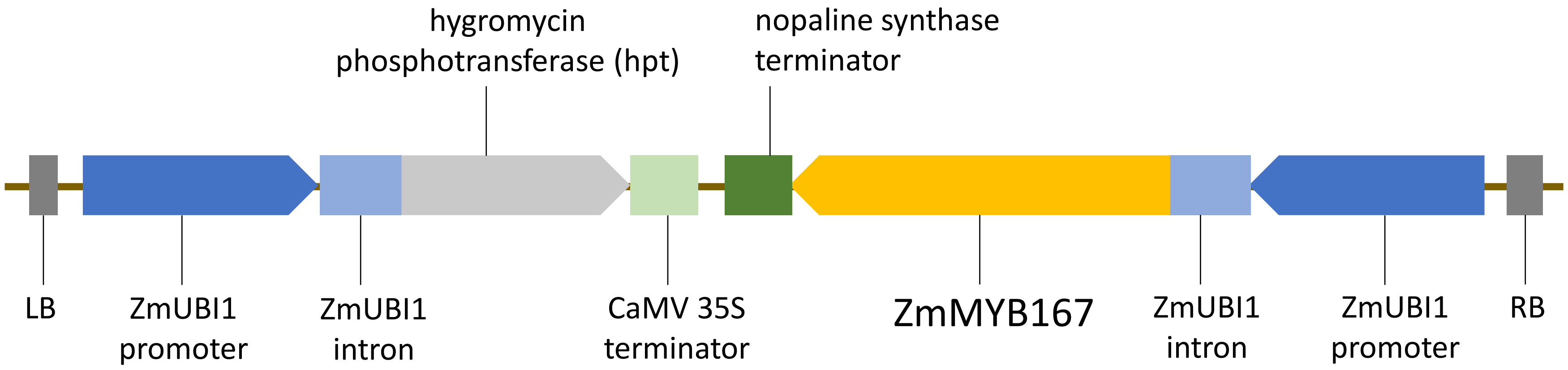

Fig. S1 Schematic representation of the genes between the left border (LB) and right border (RB) of the pIPKb002 based binary vector construct used for *Agrobacterium*-mediated transformation of *Miscanthus sinensis*.

Table S1 Cellulose and lignin-related properties in the wild-type and *ZmMYB167* transgenic *Miscanthus* plants.

Miscanthus	**LOI (A1423/A897)**	**HBI (A3400/A1320)**	**CLL (A1508/A1600)**
WT	0.59 ± 0.02	1.16 ± 0.01	0.88 ± 0.03
MS12	0.63 ± 0.01**	1.09 ± 0.02***	0.93 ± 0.03*
MS17	0.63 ± 0.02*	1.14 ± 0.04	0.92 ± 0.02*
MS18	0.64 ± 0.01**	1.12 ± 0.07	0.90 ± 0.03
MS21	0.64 ± 0.01**	1.15 ± 0.03	0.90 ± 0.02

LOI, lateral order index; HBI, hydrogen bond intensity; CLL, cross-linked lignin. Data are expressed as means ± standard deviation (n ≥ 3). Student’s t-test (two-tail): *P ≤ 0.05; **P ≤ 0.01; ***P ≤ 0.001.

Table S2 Sequences of primers used for detection of genomic DNA and reverse transcription PCR analysis.

Primer	Accession number/ Contig number	Sequences of gene-specific primers for PCR detection	Amplicon size (bp)
ZmUbi1 promoter<br>*ZmMYB167* exon	EU161568<br>GRMZM2G037650	F:5‘-TGCAGCATCTATTCATATGCTCT-3‘<br>R:5‘-CCACTGACAAGTCGGTCTCC-3‘	870
ZmMYB167	GRMZM2G037650	F:5‘-GCTGGGTTTTATGAGCAACGG-3‘<br>R:5‘-TGAATCGTCCACCAAGTTGG-3‘	130
Spermine synthase1	GRMZM2G047867<br>LOC732743	F:5‘-CTTCATGTTGGTGACGCTGT-3‘<br>R:5‘-GCCCTATTGGGTCTGATGAA-3‘	91
MsSCM3	KY930622	F:5‘-AGACCTGTCGATGGATGAGC-3‘<br>R:5‘-CGTGGAACATTGAATTGTCG-3‘	164
